# Efficacy and Day 7 Plasma Piperaquine Concentrations in African Children Treated for Uncomplicated Malaria with Dihydroartemisinin-Piperaquine

**DOI:** 10.1371/journal.pone.0103200

**Published:** 2014-08-18

**Authors:** Issaka Zongo, Fabrice A. Somé, Serge A. M. Somda, Sunil Parikh, Noel Rouamba, Philip J. Rosenthal, Joel Tarning, Niklas Lindegardh, François Nosten, Jean Bosco Ouédraogo

**Affiliations:** 1 Direction Régionale de l'Ouest, Institut de Recherche en Sciences de la Santé, Bobo-Dioulasso, Burkina Faso; 2 Non Transmissible disease department, Centre Muraz Bobo-Dioulasso, Bobo-Dioulasso, Burkina Faso; 3 Department of Medicine, Yale University School of Public Health, New Haven, Connecticut, United States of America; 4 Department of Medicine, University of California San Francisco, San Francisco, California, United States of America; 5 Mahidol-Oxford Tropical Medicine Research Unit, Faculty of Tropical Medicine, Mahidol University, Bangkok, Thailand; 6 Shoklo Malaria Research Unit, Mahidol-Oxford Tropical Medicine Research Unit, Faculty of Tropical Medicine, Mahidol University, Mae Sot, Thailand; 7 Centre for Tropical Medicine, Nuffield Department of Clinical Medicine, University of Oxford, Oxford, United Kingdom; Kenya Medical Research Institute - Wellcome Trust Research Programme, Kenya

## Abstract

**Background:**

One promising new Artemisinin-based combination therapies (ACTs) is dihydroartemisinin-piperaquine (DHA-PQ). However, the pharmacokinetics of piperaquine and the relationship between drug levels and clinical efficacy are incompletely characterized, particularly in children.

**Methods:**

We performed a single-arm open-label trial in Bobo-Dioulasso, Burkina Faso. A total of 379 participants aged 6 months or more with uncomplicated falciparum malaria were enrolled. Each participant received daily dose of DHA-PQ for three days and followed for 42 days. Parasitological efficacy was analyzed, considering rates of recrudescence and overall recurrence. PK was an exploratory endpoint and a priori, no sample size had been determined. Day 7 capillary and venous plasma concentrations of piperaquine were measured in children aged 2–10 years.

**Results:**

Of the 379 participants, 365 (96.3%) completed 42 days of follow-up. The median daily dose of PQ was 18.5 mg/kg [6.5–24]. Treatment with DHA-PQ was well tolerated with fever and parasitemia resolution within 48 hours in nearly all children. Recurrent malaria within 42 days of follow-up occurred in 31.3% (10/34) of children less than 2 years old, 16.0% (16/106) of those aged 2–5 years, 9.4% (15/160) of those aged 5–10 years, and none (0/68) of those over 10 years old. After genotyping, 3 of 41 recurrent episodes were recrudescence. An exploratory analysis shows that children with successful treatment outcomes had significantly higher median plasma concentrations of PQ compared to those with recurrent malaria within 42 days after therapy, considering either capillary samples (68 ng/ml [50–85] compared to 48 ng/ml [36–55], p<0.001) or venous samples (42 ng/ml [29–59] compared to 25 ng/ml [19–44], p<0.001).

**Conclusion:**

DHA-PQ was effective for uncomplicated P. falciparum malaria treatment and offers an alternative to other ACTs. Recurrent malaria was mainly due to new infections after treatment and was correlated with low day 7 PQ concentration in the youngest patients.

**Trial Registration:**

Controlled-Trials.com ISRCTN59761234

## Introduction

Artemisinin-based combination therapies (ACTs) are highly effective for the treatment of uncomplicated falciparum malaria [1]. In sub-Saharan Africa, artemether-lumefantrine and artesunate-amodiaquine have been widely adopted as first-line therapy. These regimens are generally effective, although artesunate-amodiaquine had lower efficacy in some studies and treatment with artemether-lumefantrine, which must be dosed twice daily and optimally with fat, is followed by high rates of new infections in areas of high malaria transmission [2], [3], [4], [5]. Among other ACTs recommended by the World Health Organization, artesunate-sulfadoxine-pyrimethamine has unacceptably poor efficacy due to resistance to sulfadoxine-pyrimethamine in many areas and artesunate-mefloquine is less well tolerated than other regimens [6], [7]. Another alternative ACT for the treatment of uncomplicated malaria in Africa is dihydroartemisinin-piperaquine (DHA-PQ). Recent studies have reported excellent efficacy and good tolerability of DHA-PQ when used for the treatment of uncomplicated malaria in Africa, with the advantage compared to artemether-lumefantrine of once daily dosing [8], [9]. PQ has a half-life of 21–28 days, which is longer than that of lumefantrine (3–4 days), the active amodiaquine metabolite desethylamodiaquine (12 days) or mefloquine (9–15 days [10], [11], [12], [13], [14], [15], [16], [17], [18]. Long half-life drugs provide longer post-treatment prophylactic effects. Nonetheless, new infections have been seen within a few weeks of therapy with DHA-PQ, due perhaps to the high variability in PQ exposure. In particular, children 2–10 years of age have lower PQ exposures because of higher body weight normalised clearance. This results in lower day 7 PQ concentration compared to older individuals, after standard weight-based dosing, causing an increased risk of recurrent parasitemia [18], [19]. To further characterise the impact of pharmacokinetics on DHA-PQ efficacy, we evaluated the efficacy of the drug to treat uncomplicated malaria in children in Burkina Faso and assessed associations between day 7 PQ plasma concentrations and treatment outcomes.

## Materials and Methods

### Ethics statement

The protocol was approved by the institutional Ethics Committee of Centre Muraz and the Committee on Human Research of the University of California, San Francisco. Written Informed consent for all participants was obtained from their parents or legal guardians prior to enrolment. The study was conducted in accordance with Good Clinical Practice, the declaration of Helsinki, the approved protocol and local regulations. The trial was registered in 2007 (N°ISRCTN59761234). The protocol for this trial and supporting CONSORT checklist are available as supporting information; see Checklist S1 and Protocol S1.

### Study site and design

This was an open-label trial in three public health centres (Colsama, Ouezzin-ville and Sarfalao) in Bobo-Dioulasso, Burkina Faso conducted from August to December 2007.Malaria is holo-endemic in the study area with peak transmission from August to October; almost all infections are due to *Plasmodium falciparum*. Within the main trial of 379 participants, 210 children 2–10 years were enrolled into a population pharmacokinetic exploratory study. The full pharmacokinetics profile of PQ in these children with uncomplicated malaria has been published elsewhere [18]. The present report describes the relationship between day 7 PQ concentrations and parasitological outcomes.

Patients were selected from the outpatient departments of the indicated health centres. Patients age 6 months or older were screened against the inclusion criteria following WHO recommendations [20]: 1) provision of written informed consent, 2) weight >5 kg, 3) history of fever in the past 24 hours or axillary temperature >37.5°C, 4) willingness to comply with 42 days of follow-up, 5) absence of known history of serious side effects from study medications, 6) no evidence of a concomitant febrile illness, 7) no history of treatment with any antimalarial in the past 2 weeks, 8) no danger signs (lethargy, unable to drink or breast feed, repeated vomiting, unable to stand/sit due to weakness) or evidence of severe malaria [1], 9) *P. falciparum* mono-infection with parasite density 2,000–200,000 parasites/µl blood; and 10) haemoglobin >5.0 g/dL. Patients who satisfied these inclusion criteria were recruited; those excluded were referred to the health centre staff for care.

### Study treatment

Enrolled participants received DHA-PQ (Holleypharm Cotec Beijing Limited, China) administered once daily for 3 days in tablets containing 40 mg of DHA and 320 mg of PQ phosphate. Children were weighed to determine the dosage, which was rounded to the nearest ¼ tablet. The target daily dosages (range) of DHA and PQ phosphate were 2.1 mg/kg (1.7–3.1) and 18 mg/kg (13.3–24.6), respectively. Treatment was directly observed. The study nurses administered all treatments in the clinic with water (no fat was given) and the patients were observed for 30 minutes to ensure that the medications were not vomited. Patients who vomited within 30 minutes of administration were retreated with a second dose at half the original dosage. Any patient who vomited more than once was excluded and referred for treatment with parenteral quinine. Paracetamol (10 mg/kg every 8 hours) was provided for treatment of febrile symptoms. Children with haemoglobin <10 g/dL were treated according to Integrated Management of Childhood Illness guidelines.

### Follow-up procedures

Patients were asked to return to the health centre for follow up on days 1, 2, 3, 4 and 7, and then weekly for 5 additional weeks after treatment and at any time they were unwell. Each visit included completion of a standardized history form, a physical examination, and, except on day 1, a finger prick for thin and thick blood smear and storage of blood on filter paper for molecular analysis. On day 7, 200 µl of capillary blood obtained by finger prick and 2 ml of venous blood were collected. During all visits, any signs or symptoms were recorded. Patients with evidence of severe malaria or danger signs or with treatment failure were referred for treatment with quinine.

### Laboratory procedures

#### Diagnosis of malaria and measurement of haemoglobin

Blood smears were stained with 10% Giemsa for 10 minutes. Parasite densities were calculated from thick smears as the number of asexual parasites per 200 leukocytes (or per 500 leukocytes if the parasite density was <10 parasites per 200 leukocytes), assuming a leukocyte count of 8000/ml. Smears were considered negative when examination of 100 high-power fields did not reveal parasites. Thin blood smears were used for parasite speciation and for gametocyte counts as the number of sexual parasites per 2000 leucocytes. Counts were performed by two technicians; discrepant readings were resolved by a third reader. Haemoglobin was measured at enrolment and on the last day the patient was seen using a portable spectrophotometer (HemoCue, Ängelhom, Sweden).

#### Measurement of PQ concentrations

All capillary and venous blood samples were centrifuged at 2000 g for 10 minutes, and plasma was separated and frozen at −20°C until shipment on dry ice to the Department of Clinical Pharmacology, Bangkok, Thailand. Plasma concentrations of PQ were quantified by a validated liquid chromatographic tandem mass-spectrometric method [21]. For quality control standards at 4.5, 20 and 400 ng/ml were analysed with each batch to ensure precision of the assay. The standard deviation of quality control samples was below 5%, indicating excellent assay performance. The lower limit of quantification was set to 1.50 ng/ml. The Department of Clinical Pharmacology is participating in the World Wide Antimalarial Resistance Network quality control and assurance proficiency testing programme (http:/www.wwarn.org/toolkit/gaqc).

#### Molecular analysis

Whenever blood was collected, 3–4 drops were placed onto filter paper (Whatman 3MM), labelled, air-dried, and stored in sealed plastic bags at ambient temperature. Parasite DNA was subsequently extracted using the Chelex method [22]. For patients who experienced recurrent malaria after day 6, the samples collected at baseline and at the time of recurrence were genotyped in a stepwise fashion using msp-2, msp-1, and six microsatellites [23]. If, for any of the loci, an allele was not shared between day 0 and the day of recurrence, the infection was classified as a new infection. If at least one allele was shared between day 0 and the day of recurrence for each locus, the infection was classified as a recrudescence.

#### Study endpoints

The primary endpoint was treatment efficacy (adjusted by genotyping and unadjusted) over 42 days. Outcomes were classified as: 1) early treatment failure (danger signs, complicated malaria, or failure to adequately respond to therapy between days 0 and 3), 2) late clinical failure (danger signs, complicated malaria, or fever and any parasitemia between days 4 and 42), 3) late parasitological failure (any recurrence of parasitemia between days 4 and 42 without fever (axillary temperature ≥37.5°C or history of fever in the past 24 hours) and 4) adequate clinical and parasitological response (no parasitemia over 42 days of follow-up) [20].

Secondary outcomes included the resolution of fever and parasite clearance over the first three days after treatment, the change in haemoglobin level over time, the occurrence of gametocytes, adverse events of any grade during follow-up, and the correlation of day 7 concentrations of PQ and efficacy. Patients were excluded from the efficacy assessment if they used antimalarial drugs outside of the study, if they experienced serious adverse events requiring discontinuation of treatment, or if they withdrew informed consent.

#### Sample size and statistical analysis

Based on a trial in which we reported day 42 unadjusted treatment efficacy of 92.5% for DHA-PQ, we determined that we needed to enrol 379 patients to demonstrate this efficacy with a precision of 5.5%, 80% power and 5% significance level, accounting for a predicted 7.5% loss to follow-up. PK was an exploratory endpoint and a priori, no sample size had been determined. Data were single entered using EPI INFO 6.01 and analysed with STATA 11.0 (STATA Corporation College Station, TX, USA). Efficacy outcomes were analysed by intention to treat and per protocol. Data for patients who were lost to follow-up without a defined primary efficacy outcome were included in the analyses until the last day seen. Day 7 PQ concentration cut-offs of 30 ng/ml for venous samples and 57 ng/ml for capillary samples were used in this analysis, as levels below these cut-offs have been found to predict a higher risk of recrudescent malaria, compared to greater levels, in previous studies [18]. The chi-square test or Fisher's exact test were used to compare proportions. Normally distributed or log-transformed (when not meeting the normality assumption) continuous variables were compared using the Student's t-test (paired or unpaired). Statistical significance was defined as a p-value below 0.05. We used the Kaplan Meier survival method to estimate the risk of recurrent parasitemia over the period of follow up; patients were censored at the time of withdrawal or recurrent parasitemia. A Cox regression model was used to estimate the hazard ratio of malaria; parameters included in the model were age, weight at entry and day 7 piperaquine concentrations.

## Results

### Study profile and baseline characteristics

**Figure 1:**
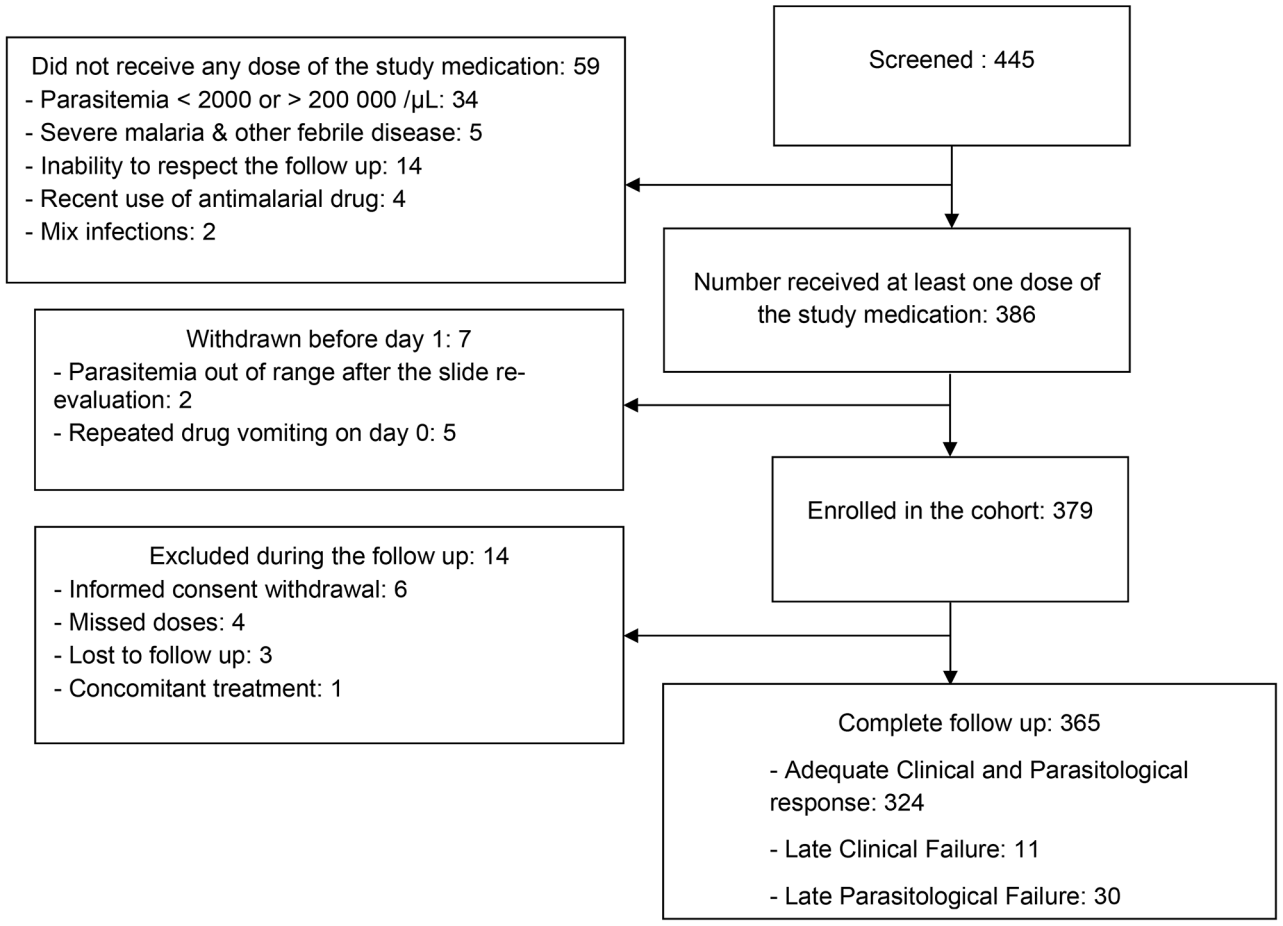
Trial profile.

Of 445 patients diagnosed with malaria and screened for enrolment, 386 met entry criteria and were enrolled in the study (Figure 1). The main reason for exclusion was parasitemia <2,000/µl or ≥200,000/µl. Seven participants withdrew on the first day of the study, primarily due to persistent vomiting. Of the 379 enrolled participants who remained in the study on day 1, 96.3% (365/379) completed 42 days of follow-up. The median (range) age of participants was 6 years (0.6–50); 36% of subjects were under 5 years of age. The median (range) daily administered dose of PQ phosphate was 18.5 mg/kg (6.5–24). The baseline characteristics of study subjects are summarised in Table 1. The population pharmacokinetic study enrolled 226 children 2–10 years of age; this sub-sample provided 226 capillary and 198 venous samples on day 7.

**Table 1:** Characteristics of the enrolled patients at baseline.

Parameters		Age categories				
		<2 years (n = 34)	2–4 Years (n = 112)	5–10 Years (n = 165)	>10 years (n = 68)	Total (N = 379)
Sites	Colsama	8	38	64	43	153
	Ouezzin-Ville	23	41	51	16	131
	Sarfalao	3	33	50	9	95
Gender (Male %)		15 (44.1%)	62 (55.4%)	93 (56.4%)	26 (38.2%)	196 (51.7%)
Weight in kg, 95% CI		9.1 (8.6–9.5)	13 (12.5–13.5)	21.3 (20.6–22)	44.5 (40.2–48.8)	21.9 (20.5–23.4)
Temperature, 95% CI		38.8 (38.4–39.1)	38.8 (38.6–38.9)	38.5 (38.4–38.7)	38.1 (37.8–38.3	38.5 (38.4–38.6)
Mean parasite count (geometric), 95% CI		22657 (14734–34840)	30200 (24699–36926)	26054 (22083–30740)	17925 (13765–23342)	25133 (22483–28095)
Heamoglobin g/dL, arithmetic mean (SD)		8.8 (8.1–9.6)	9.6 (9.3–10)	10.8 (10.5–11.1)	11.9 (11.5–12.4)	10.5 (10.3–10.7)

### Treatment efficacy

Over 42 days of follow up, recurrent parasitemia occurred in 31.3% (10/32) of children less than 2 years old compared to 16% (16/106) in those aged 2–4 years (p = 0.057), 9.4% (15/160) in those aged 5–10 years (p<0.001) and 0% (0/68) in those aged over 10 years (X^2^ test for linear trend = 23.85, p<0.001; Table 2). Of the enrolled patients, 5.3% (20/379) received a daily dose of piperaquine less than 13.3 mg/kg per day (under dose) and 0.3% (1/379) a child aged 25 months received higher dosage (>24.6 mg/kg per day). Out of the patients who completed the follow up with assigned clinical outcome, 19 patients were under dosed but presented successful clinical outcome and one (1) child aged 25 months despite he has been over dosed presented a late parasitological failure.

**Table 2:** Treatment outcome stratified by age groups.

	<2 years	2–4 years	5–10 years	>10 years	Total
Category size	32	106	160	67	365
Median PQ dose in mg/kg^−1^ daily (range)	17.8 (15.2–24)	18.9 (11.4–21.8)	18.8 (14.2–24)	16.8 (6.5–19.3)	18.5 (6.5–24)
Early treatment failure	0	0	0	0	0
Late clinical failure, 95% CI	6 (18.7%) [5.2–32.2]	2 (1.9%) [−0.7–4.5]	3 (1.9%) [−2%–4]	0	11 (3%) [12–4.8]
Late parasitological failure, 95% CI	4 (12.5%) [1]–[24]	15 (14.1%), [7.5–20.7]	11 (6.9%) [−7.1%–21]	0	30 (8.2%) [5.4–11]
Total number recurrence	10	17*	14	0	41
Recrudescence	2	1	0	0	3
New infections	8	15	14	0	37
Adequate clinical and parasitological response, 95% CI	22 (68.7%) [52.6–85]	89 (84%) [77–91])	146 (91.2%) [86.8–95.6]	67 (100%)	324 (88.8%) [85.6–92]

**Figure 2:**
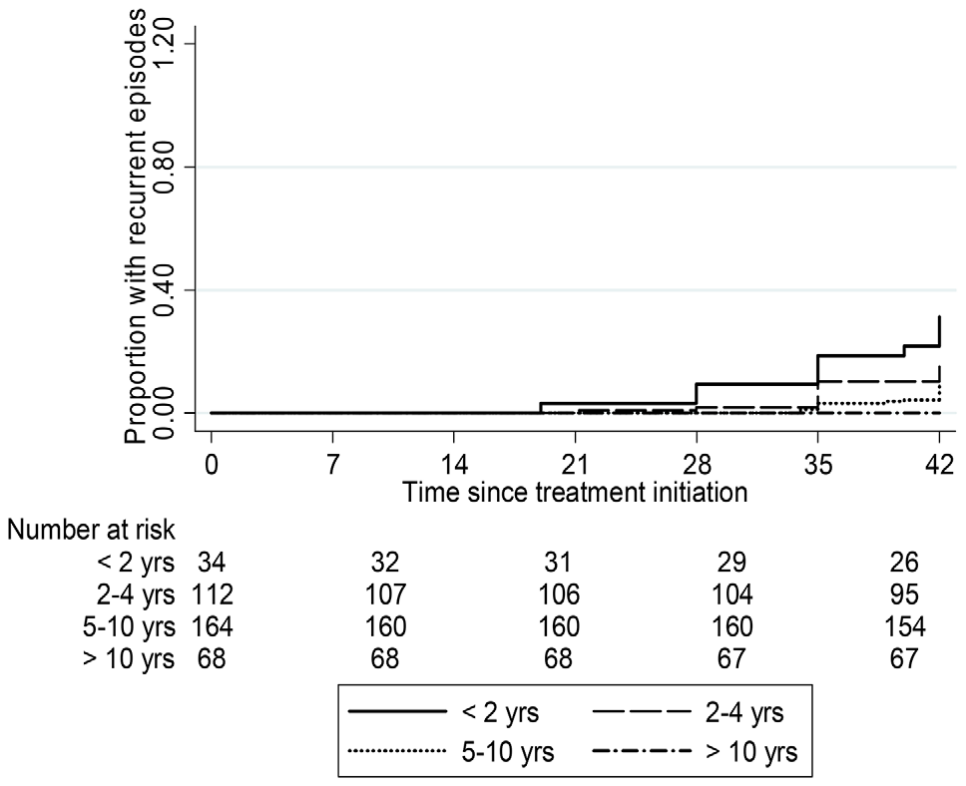
Kaplan-Meier estimate of proportion with recurrent parasitemia per age categories.

In the patients with recurrent malaria, the time to recurrence was 19 days in those under 2 years of age, 21 days in those 2–4 years old (p = 0.6) and 34 days in those 5–10 years old (p = 0.01) (Figure 2).

When the rate ratio for malaria (treatment failure) was estimated using the Cox regression model, the hazard ratio (HR) decreased as the age increased. Compared to children <2 years old, the HR was 0.49 (95% CI 0.23–1.03, p = 0.06) for those 2–5 years and 0.15 (95% CI[0.06–0.36] p<0.001) for those more than 5 years old. Likewise the HR was significantly higher in patients with day 7 capillary piperaquine concentration below the cut off of 57 ng/ml, compared to those with levels above this value (HR 0.24, 95% CI[0.11–.52], p<0.001) and in patients with low venous concentrations of piperaquine (HR 0.30, 95% CI[0.13–0.70], p = 0.006). Patients with body weight below the median value (18 kg) had a higher hazard ratio (HR 0.25, 95% CI 0.12–0.51], p<0.001) and each unit (kg) increase in body weight reduced the hazard ratio by 14%, 95% CI [8.9%–19%]. After genotyping, 37 of 41 episodes were classified as new infections, 3 as recrudescent infections, and one was indeterminate (Table 2). The 3 recrudescent cases occurred in two 14 month old children and one 36 month old child, and presented on days 19, 21, and 42 days of follow-up.

### Association between plasma PQ concentrations and treatment outcomes

To evaluate the association between day 7 levels of PQ and treatment outcomes, we conducted a sub-study in a population of 226 children 2–10 years.

**Figure 3:**
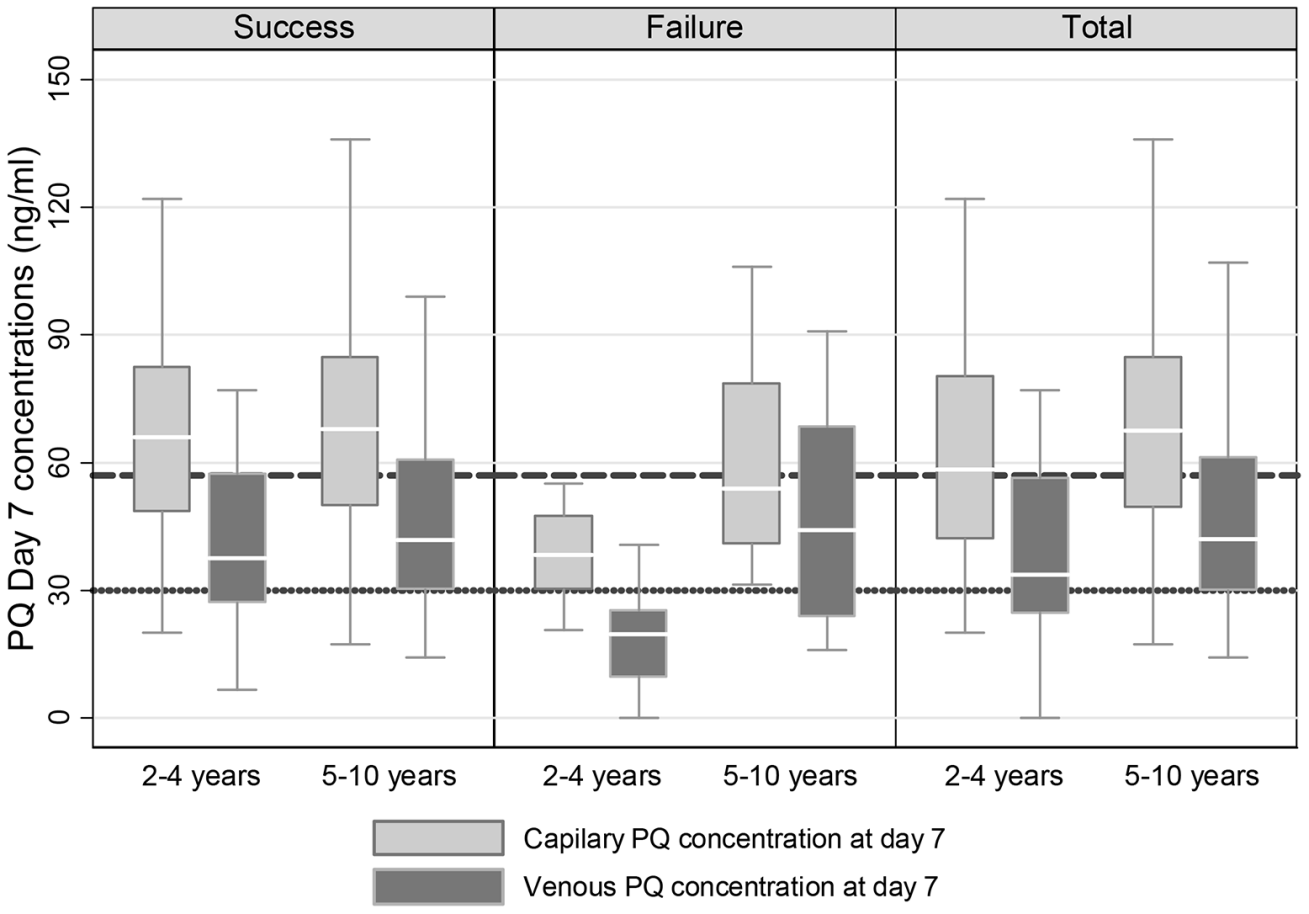
Box plot for the distribution of PQ concentration by treatment outcome. The dotted lines represent a previously proposed cut-off for piperaquine concentrations quantified in venous (30 ng/ml) and capillary (57 ng/ml) blood samples (Tarning et al. 2012).

In total 226 capillary and 198 venous plasma samples were obtained on day 7 from this population (children 2–10 years old). Median [range] day 7 PQ concentrations were significantly higher in capillary samples (67 ng/ml [49–84]) compared to venous samples (41 ng/ml [27–59]), p<0.001 (Table 3). Children under 5 years of age, as compared to older children, had significantly lower median day 7 levels in venous blood (p = 0.03), but not in capillary blood (p = 0.26) (Table 3 and Figure 3). Children with successful treatment outcomes had significantly higher (p<0.001) median [range] plasma concentrations of PQ, compared to those with recurrent malaria, considering either capillary samples (68 ng/ml [50–85]) in successful outcomes and 48 ng/ml [36–55] in children with recurrent malaria) or venous samples (42 ng/ml [29–59] in successful outcomes and 25 ng/ml [19–44] in children with recurrent malaria) (table 4).

**Table 3:** Capillary and venous concentrations of PQ by age.

[PQ] ng/ml	2–5 years, n = 60	6–10 years, n = 126	Total, n = 186	P-value
Capillary sample	62.2 [45.4–83.3]	67.50 [49.6–84.5]	66.70 [48.5–84.4]	0.23
Venus sample	34.00 [24.6–56.8]	41.90 [29.8–63.5]	40.95 [27.3–58.9]	0.02
P-value	<0.001	<0.001	<0.001	

Median values [interquartile ranges] are shown; Wilcoxon Rank Sum Test; Wilcoxon Paired Sign Rank Test.

**Table 4:** Plasma concentrations of PQ and treatment outcomes among children 2–10 years.

[PQ] ng/ml	Success, n = 165	Failure, n = 21	Total, n = 186	P-value
Capillary sample	67.80 [50.4–84.8]	47.50 [36–55.4]	66.70 [48.5–84.4]	<0.001
Venus sample	41.60 [29.1–59.4]	25.30 [18.6–44.2]	40.95 [27.3–58.9]	0.010
P-value	<0.001	<0.001	<0.001	

Success represents adequate clinical and parasitological response; failure represents recurrent parasitemia.

### Fever and parasite clearance, gametocyte carriage and mean haemoglobin change

Parasite clearance was rapid, with parasites cleared within 2 days of initiation of therapy in 98% of episodes (table 5). Over 90% of the patients cleared fever by day one after treatment initiation, and over 99% by day 2 (table 5). Seven patients had gametocytes at enrolment: 3 cleared the gametocytes by day 2, 3 others by day 21 and the last by day 28. All of them remained free of gametocytes up to the end of follow up. The prevalence of gametocytes in the patients free of gametocytes on day 0 was 6%, 4%, 3%, and 2% on day 2, 3, 7, and 14, respectively. Less than 1% had gametocytes from day 21 to 35, and all were free on day 42. Haemoglobin levels increased between day 0 and the last day of follow up; the mean gain was 1.2 g/dL (95%CI [1.0–1.4], p<0.001) and was significantly higher in the patients with an adequate clinical and parasitological response compared to those with recurrent infection (11.9 g/dL versus 10.9 g/dL, mean difference of 0.9 g/dL, 95%CI [0.4–1.5], p = 0.001).

**Table 5:** Proportion of patients who cleared fever.

Days after initiation of treatment	Number with cleared fever	Proportion	Days after initiation of treatment	No. with cleared parasitemia	Proportion
Day 1 (n = 357)	355	93.8%	Day 2 (n = 350)	325	92.9%
Day 2 (n = 352)	344	97.7%	Day 3 (n = 349)	346	99.1%
Day 3 (n = 351)	345	98.1%	Day 4 (n = 227)	226	99.6%

### Treatment acceptability and safety

DHA-PQ was well tolerated. The most common recorded adverse events were vomiting, headache and diarrhoea (table 6). Vomiting within 30 minutes occurred during treatment administration in 5.1% (19/376) of subjects and was not correlated with the dose received. Day 7 PQ concentration was similar in children who vomited within thirty minutes and those who did not. No severe adverse events requiring the discontinuation of treatment were recorded.

**Table 6:** Proportion of patients with adverse events of any grade.

Signs/symptoms	Frequency	Percentage
Vomiting within 30 minutes (n = 354)	18	5.1%
Headache (n = 278)	15	5.4%
Vomiting (n = 355)	22	6.2%
Diarrhea (n = 357)	12	3.4%
Anorexia (n = 357)	11	3.1%
Weakness (n = 357)	9	2.5%
Pruritus (n = 356)	9	2.5%
Nausea (n = 294)	4	1.4%

## Discussion

The objectives of this study were to evaluate the efficacy of DHA-PQ for the treatment of uncomplicated falciparum malaria and, in children, to assess associations between day 7 plasma concentrations of PQ and treatment outcomes. DHA-PQ demonstrated excellent treatment efficacy, with good tolerability and safety, although treatment failures, generally due to new infections over 42 days after therapy, were seen in 20% of children less than 5 years of age. In children 2–10 years of age, day 7 PQ concentrations were associated with recurrent malaria after therapy. Children with a day 7 capillary PQ concentration below 57 ng/ml, a previously identified efficacy cut-off, had a higher risk of parasitological recurrence compared to those with a concentration above this threshold [24]. Thus, low blood concentrations of PQ in younger children were associated with inferior treatment efficacy.

Our results support previous reports demonstrating excellent efficacy for DHA-PQ at the same study site and in other parts of Africa [7], [9], [25], [26], [27]. DHA-PQ has important advantages over other ACTs. First, it is likely better tolerated than some ACTs, in particular artesunate-amodiaquine and artesunate-mefloquine, although head-to-head comparisons of these regimens in African children are not available. Second, DHA-PQ offers once daily dosing, simplifying treatment compared to artemether-lumefantrine, which must be administered twice-daily with fat [28], [29]. Third, due to the long half-life of PQ, DHA-PQ offers a prolonged post-treatment prophylactic effect, translating into lower rates of recurrent malaria compared to other ACTs [25], [29]. Considering all these factors, DHA-PQ may be the optimal ACT for the treatment of uncomplicated falciparum malaria in areas of high transmission intensity.

Genotyping to compare initial and recurrent isolates identified only 3 episodes of recrudescence. Thus, the antimalarial efficacy of DHA-PQ was excellent. However, recurrences within 42 days after therapy were common, especially in young children. The benefits of DHA-PQ must be balanced against potential selection by PQ of parasites resistant to this drug. Importantly, resistance to PQ was described some decades ago in China [30]. Although recent definitive evidence of resistance to PQ has not been reported in studies from Africa or Asia, *ex vivo* sensitivity to the drug has varied considerably, and careful continued surveillance of parasite susceptibility is important [31], [32], [33]. Reassuringly, treatment with DHA-PQ did not select for parasite polymorphisms that are associated with resistance to the related aminoquinolines chloroquine and amodiaquine [34].

DHA-PQ was well tolerated, as reported in previous studies [35], [36]. However, 5.1% of patients vomited the drug within 30 minutes after administration. Such information is not usually reported in the assessment of drug tolerability, but vomiting after treatment with DHA-PQ has been noted in other trials [35]. The immediate tolerability of a drug is a key factor for acceptability, especially in children, and it is unclear if vomiting will limit acceptability of DHA-PQ in African children.

## Conclusion

DHA-PQ demonstrated excellent efficacy and was well tolerated in treating uncomplicated falciparum malaria in African children. The regimen is a good alternative to other ACTs for the treatment of uncomplicated malaria. However, in children recurrent infections were common after treatment and associated with low PQ drug concentrations, suggesting that current dosage recommendations for DHA-PQ in young children need re-evaluation to provide optimum post-exposure prophylaxis in this vulnerable group.

## Supporting Information

Table S1 **Day 7 Concentration of Piperaquine and Vomiting within 30 Minutes following the Treatment Administration.**
(DOC)

Checklist S1 **CONSORT Checklist.**
(DOC)

Protocol S1 **Day 7 Plasma Piperaquine Concentrations and Dihydroartemisinin-Piperaquine Efficacy in Children Treated for Uncomplicated Malaria in Burkina Faso.**
(DOC)
