# Supplementary material for: Efficacy and Day 7 Plasma Piperaquine Concentrations in African Children Treated for Uncomplicated Malaria with Dihydroartemisinin-Piperaquine
Source: PLoS One. 2014 Aug 18;9(8):e103200. doi: 10.1371/journal.pone.0103200 (PMC4136730; doi:10.1371/journal.pone.0103200)
Supplement: Table S1 — Day 7 Concentration of Piperaquine and Vomiting within 30 Minutes following the Treatment Administration. (DOC) [file pone.0103200.s001.doc]

Table S1. Day 7 concentration of Piperaquine and vomiting within 30 minutes following the treatment administration

|  |  | Vomiting within 30 minutes | |  |
| --- | --- | --- | --- | --- |
|  |  | Yes | No | Total |
| Piperaquine concentration on day 7 | < 57.0 ng/ml | 1 | 79 | 80 |
|  | >=57.0 ng/ml | 7 | 122 | 129 |
|  | Total | 8 | 201 | 209 |
