# Supplementary material for: Efficacy and Day 7 Plasma Piperaquine Concentrations in African Children Treated for Uncomplicated Malaria with Dihydroartemisinin-Piperaquine
Source: PLoS One. 2014 Aug 18;9(8):e103200. doi: 10.1371/journal.pone.0103200 (PMC4136730; doi:10.1371/journal.pone.0103200)
Supplement: Protocol S1 — Day 7 Plasma Piperaquine Concentrations and Dihydroartemisinin-Piperaquine Efficacy in Children Treated for Uncomplicated Malaria in Burkina Faso. (DOC) [file pone.0103200.s003.doc]

**Pharmacokinetics of Dihydroartemisinin-Piperaquine in the treatment of uncomplicated falciparum malaria in children in Burkina Faso**

Version 1.0, May 07

**Statement**

Artemisinin-based combination therapies (ACTs) have recently been adopted by National Malaria Control Programmes in Africa as first line therapies to treat uncomplicated malaria, but the optimal choice of ACT remains uncertain. . Dihydroartemisinin-piperaquine (DP) is a promising candidate for first-line therapy of malaria. Preliminary results of pharmacokinetic studies indicate that the disposition of piperaquine is altered in children and this fact may affect the efficacy of the treatment. This project will evaluate the efficacy and the pharmacokinetics of DP in order to design a “best regimen” for the treatment of malaria in children.

**BACKGROUND**

Malaria endemic countries are facing the challenge posed by the wide spread of drug resistance[1, 2], which requires the implementation of alternative therapies.

Combination therapies, particularly ACTs, are advocated as the best way to improve drug efficacy while slowing the extension of the resistance. [3, 4]

Many countries in Africa[5-7], including Burkina Faso, have moved to artemether-lumefantrine (Coartem®, AL) or Artesunate/amodiaquine (AS/AQ) as first-line or second line in the treatment of malaria in a number of other countries [8]. In recent studies in Bobo-Dioulasso, Burkina Faso [5-7, 9], the efficacy against uncomplicated malaria of AL, considering both recrudescent and new infections after therapy, was significantly worse than that of DP (Zongo, et al., submitted). DP has also shown promising results in studies in Asia and Africa [10, 11] . However, DP has not been as extensively studied as other ACTs, and our understanding of its pharmacokinetics are incomplete. Indeed, this drug appears to have modified kinetics in infants and adults [12] which may seriously reduce its efficacy.

Therefore, we aim to study the pharmacokinetics properties of DP in children to provide evidence based data to allow optimal treatment of uncomplicated falciparum malaria in children.

**Goal**

To evaluate the efficacy and pharmacokinetics of DP for designing an optimal regimen for its use in children.

**Objectives**

1. To characterize the pharmacokinetics of piperaquine in children
2. To determine the efficacy and safety of DP for the treatment of uncomplicated falciparum malaria in Burkina Faso.
3. To search for associations between DP treatment outcomes and pharmacokinetics in individual patients.

**Study design**

1. Treatment efficacy

This will be an open-label trial in Bobo-Dioulasso, designed in accordance with WHO guidelines[13]. The target populations include residents of the catchments areas of the Colsama, Sarfalao, and Ouezzin-ville dispensaries in Bobo-Dioulasso. Children who present to the study clinics with symptoms suggestive of malaria will be screened with a thick blood smear. Subjects who meet selection criteria of treatment efficacy will be treated and followed up for 42 days. Efficacy outcomes will be assessed based on the 2006 WHO outcomes classification system [13].

2. Pharmacokinetic analysis

Pharmacokinetic assays and analysis will be performed at the Wellcome Trust Laboratory in Bangkok in collaboration with one of our staff in Bobo-Dioulasso and at UCSF.

Population kinetic studies using sparse sampling and capillary sampling are proposed to determine if the dosing of DP needs to be modified in young children. Sparse capillary sampling at day 0, 2 (2 hrs post dose), 4, 5, 7, 14, 21, 28, 35, 42 will be used to describe the kinetics of piperaquine. The sampling method is critical: capillary blood (200 µL) will be taken and plasma separated (centrifugation at 3000 g for 10 mn) and frozen at -20C. The assays and the analysis will be performed by the pharmacology laboratory in Bangkok (N.Lindegardh and J. Tarning). The exact time for sampling will be randomly allocated and scattered within the days indicated above. The clock hour at the time of sampling and dosing must be recorded accurately. It is important that the blood is obtained without squeezing the finger tip, as this will dilute the blood in interstitial fluids. Also important is to avoid any hemolysis, as this will interfere with the assay.

*3. Study outcome*

Primary outcome

- PK parameters: Piperaquine concentration-time profiles will be analyzed using non-linear mixed effects modeling as implemented in NONMEM. Different compartmental models will be tried and primary pharmacokinetic parameters will be parameterized as oral clearance (CL/F), steady state volume of distribution(s) (Vss/F), inter-compartment clearance(s) (Q/F) and absorption rate (ka). Continuous and dichotomy co-variates will be investigated. Exposure-response correlations will be investigated in case of recrudescence and/or reinfection in a significant number of patients.

Secondary outcomes

- Risk of recurrent malaria
- Risk of clinical and parasitological treatment failure

Risks will be estimated using the Kaplan-Meier product limit formula based on a modified intention-to-treat analysis.

- Prevalence of fever (defined as both subjective fever in the previous 24 hours and measured axillary temperature > 37.5°C) on follow-up days 1, 2, and 3.
- Prevalence of parasitemia on follow-up days 2 and 3.
- Change in mean hemoglobin from day 0 to 42 (or day of rescue therapy for patients classified as LCF or LPF)
- Prevalence of gametocytemia on follow-up days 2, 3, 7, 14, 21 and 28.
- Change in the prevalence of molecular markers possibly associated with drug resistance on day 0 or the day of recurrent parasitemia, including polymorphisms in pfcrt and pfmdr1.
- In vitro sensitivity to antimalarial drugs.

Classification of treatment outcome

Response to treatment will be classified according to the 2006 WHO classification system with slight modifications[13] and will include adequate clinical and parasitological response (ACPR), early treatment failure (ETF), late clinical failure (LCF), and late parasitological failure (LPF)

For purposes of data reporting in the final analysis, treatment outcomes will be dichotomized based on the following definitions:

- Recurrent malaria = ETF + LCF
- Recurrent parasitemia = ETF + LCF + LPF.

All ETFs will be considered true treatment failures. For all LCFs and LPFs, molecular genotyping (based on polymorphisms in msp-1 and msp-2) will be used to distinguish recrudescence from new infection.

- Clinical treatment failure = All ETFs + LCFs due to recrudescence
- Parasitological treatment failure = All ETFs+LCFs+LPFs due to recrudescence

### *4. Assessment of adverse events*

### Definition

### An adverse event is defined as "any untoward medical occurrence in a patient or clinical investigation subject administered a pharmaceutical product that does not necessarily have a causal relationship with this treatment." (ICH Guidelines E2A). An adverse event can further be broadly defined as ANY untoward deviation from baseline health which includes:

### Worsening of conditions present at the onset of the study

### Deterioration due to the primary disease

### Intercurrent illness

### Events related or possibly related to concomitant medications

### (International Centers for Tropical Disease Research Network Investigator Manual, Monitoring and Reporting Adverse Events, 2003).

### A serious adverse event is defined as an experience that results in any of the following outcomes:

### Death during the period of study follow-up

### Life-threatening experience (one that puts a patient at immediate risk of death at the time of the event)

### Inpatient hospitalization during the period of study follow-up

### Persistent or significant disability or incapacity

### Specific medical or surgical intervention to prevent one of the other serious outcomes listed in the definition.

### Identification of adverse events:

At each follow-up visit (days 1, 2, 3, 4, 5, 7, 14, 21, 28, 35, and 42 and any unscheduled day, study clinicians will assess patients according to a standardized clinical record form. A severity grading scale, based on toxicity grading scales developed by the WHO and the National Institutes of Health, Division of Microbiology and Infectious Diseases, will be used to grade severity of all symptoms, physical exam findings, and hemoglobin results. Any new event, or any event present at baseline that is increasing in severity, will be considered an adverse event.

Reporting of adverse events:

For each possible adverse event identified and graded as moderate, severe or life threatening, an adverse event report form will be completed. An adverse event report form will not be completed for events classified as mild, as these symptoms are common and difficult to distinguish from signs and symptoms due to malaria. The following information will be recorded for all adverse experiences that are reported:

1. Description of event
2. Date of event onset
3. Date event reported
4. Maximum severity of the event
5. Maximum suspected relationship of the event to study medication
6. Is the event serious?
7. Initials of the person reporting the event
8. Was the event episodic or intermittent in nature?
9. Outcome
10. Date event resolved

Reporting of serious adverse events and protocol deviations

The IRSS core facility staff will be responsible for notifying the IRBs that a serious adverse event has occurred. All serious events shall be reported to the UCSF Committee of Human Research (CHR) within 10 days of the onset of the event. A UCSF AE report form (one for each event) shall be completed and faxed to the CHR. Any protocol deviations or problems involving study conduct or patient participation shall be reported in a similar manner, following IRB procedures.

Management of serious adverse events

For all SAEs, the relationship of the events to the study medication shall be determined by the clinicians. If a patient experiences an SAE while receiving treatment for uncomplicated malaria with their assigned study treatment, the study medication will be stopped if the SAE is felt to be probably or definitely related to the study medication and the patient will be treated with quinine. Plans for additional follow-up, including laboratory tests, will be individualized to each subject as appropriate for the clinical setting. If an SAE occurs while a patient is not receiving treatment with study medications, treatment and plans for follow up will be individualized as appropriate for the clinical setting.

**RECRUITMENT &TREATMENT PROCEDURES**

## *Study sites*

The study will be conducted in Bobo-Dioulasso, the second main city of the country. The patients will be recruited in 3 public dispensaries for children (Colsama, Ouezzin-Ville, Sarfalao)

1. ***Recruitment & selection criteria***

Study subjects will be recruited from the public health facilities. Patients who present with symptoms suggestive of malaria (fever or history of fever) will be referred to the outpatient laboratory for a fingerprick for screening thick blood smear (using standard Giemsa staining). Thick blood slides will be read and counted by the laboratory technicians. The parasite density of positive screening thick blood smears will be estimated by counting the number of asexual parasites per 200 leukocytes, assuming a leukocyte count of 8,000/μl. At the time of screening, study personnel will record basic information including patient age, gender, axillary temperature and date. All patients who have a positive screening thick smear with a parasite density of 2000-200,000/ul will be referred to the study clinician for further evaluation. If the patient satisfies the selection criteria, he/she will be enrolled in the study and referred to the study nurse for treatment administration. All patients who do not satisfy the initial selection criteria and are excluded from study enrolment will be referred back to the outpatient department for appropriate care.

## Selection criteria

On day 0, patients with symptoms suggestive of malaria and a positive screening thick blood smear will be assessed for the following selection criteria by study physicians:

1. Not previously enrolled in this study
2. Age > 6 months
3. Weight > 5 kg
4. Fever (> 37.5ºC axillary) or history of fever in the previous 24 hours
5. Absence of any history of serious side effects to study medications
6. No evidence of a concomitant febrile illness in addition to malaria
7. Provision of informed consent and ability to participate in 42-day follow-up (patient has easy access to health unit)
8. No history of antimalarial use in the previous two weeks
9. No danger signs or evidence of severe malaria defined as:

- Unarousable coma (if after convulsion, > 30 min)
- Recent febrile convulsions (within 24 h)
- Altered consciousness (confusion, delirium, psychosis, coma)
- Lethargy
- Unable to drink or breast feed
- Vomiting everything
- Unable to stand/sit due to weakness
- Severe anemia (Hb < 5.0 gm/dL)
- Respiratory distress (labored breathing at rest)
- Jaundice

Patients fulfilling these criteria will be assigned a study number and will be referred to the laboratory. A second fingerprick blood sample will be obtained to prepare thick and thin blood smears, collect blood on filter paper, and for measurement of hemoglobin. Patients with hemoglobin levels < 5.0 gm/dL will be excluded after enrollment based on criteria for severe malaria (see above) and immediately referred back to the study physicians for appropriate care. After going to the laboratory, the subjects will be referred to the study nurse for treatment allocation and treatment with the study medications. Patients must also meet the following criterion:

1. Absence of repeated vomiting of study medications on day 0

Results of the Giemsa-stained thick and thin blood smears obtained on day 0 will not be available until after the patients have been treated and discharged from the clinic. Patients will return to the clinic on day 1 and will be excluded from the study if the following inclusion criteria are not met:

1. *P. falciparum* mono-infection
2. Parasite density 2000-200,000/ul
3. ***Treatment administration***

Administration of medications will be performed by the study nurse who will record the date and time of treatment and the patient's study number.

Administration of all study drugs will be directly observed. If a patient fails to return to the clinic he/she will be visited at home by a home visitor who will collect information on where patients live. If patients miss their second dose of study drug, they will be excluded from the study. Study drugs given to young children will be crushed, mixed with water, and administered as slurry. Study drugs administered to older children will be given as tablets or fractions of tablets to be taken orally with a glass of water. Patients will be observed for 30 minutes to ensure that the medications are not vomited. Any patient who vomits the medication within 30 minutes of administration will be retreated with a second dose. Any patient who vomits repeatedly (> 3 times) will be excluded and referred for treatment with parenteral quinine.

### 3.1. Study drugs

*Dihydroartemisinin piperaquine*

### DP is a fixed combination drug containing the artemisinin derivative, dihydroartemisinin, and piperaquine. Dihydroartemisinin is highly active and also the main in vivo metabolite of the drugs artesunate and artemether. Piperaquine is a bisquinoline that retains activity against chloroquine-resistant *P. falciparum*. Piperaquine replaced chloroquine as the first-line treatment for malaria in China in 1978.

### *Dosing of Dihydroartemisinin Piperaquine*

All subjects will receive DP once daily for 3 days in the morning in fixed dose tablets (40 mg dihydroartemisinin + 320 mg piperaquine) according to weight-based guidelines. A weight-based regimen consisting of a total dose of 7 and 55 mg/kg of dihydroartemisinin and piperaquine, respectively, will be given in 3 equally divided daily doses. (Appendix 6)

## 3.2. Additional medications

On day 0, patients will receive paracetamol (10mg/kg) to take every 8 hours until the resolution of fever. Patients found to have uncomplicated malaria and a concomitant illness will be treated for both and followed up according to the study protocol. For patients with anemia (Hb < 10 gm/dL), we will follow Integrated Management of Childhood Illness (IMCI) guidelines: For patients with anemia (Hb < 10 gm/dL), we will follow Integrated Management of Childhood Illness (IMCI) guidelines: anemic children will be treated with iron sulfate (100 mg daily for 2 weeks) and mebendazole (only children > 1 year of age; 250 mg age 1-2 years; 500 mg > 2 years age; treated no more frequently than every 6 months).

.

# Follow up and laboratory procedures

## *1. Follow-up schedule*

Children will be asked to return to the clinic for follow-up on days 1, 2, 3, 4 , 5, 7, 14, 21, 28, 35, 42 and any unscheduled day that they feel ill. All patients will be reimbursed the cost of their transport to and from the clinic. At enrollment, details about the location of the patient’s residence will be obtained and if a subject does not return for a scheduled clinic follow-up appointment, the study personnel shall visit them at home. Patients who return on day 1 and fail to fulfill the criteria of *P. falciparum* mono-infection with a parasite density of 2000-200,000 parasites/ul will be excluded from the study and referred to the dispensary staff for management. At each repeat visit, temperature will be measured and a focused physical examination will be performed. A finger prick blood sample will be obtained on days 2, 3,4, 5, 7, 14, 21, 28, 35, 42 and any extra day they present with a fever to repeat thick blood smears and to save blood on filter paper. Hemoglobin will be re-evaluated on day 42 or at the time of clinical treatment failure (ETF or LCF).

***2. Laboratory studies***

(i) Diagnosis of malaria

Patients who present with symptoms suggestive of malaria (fever or history of

fever) will be referred to the outpatient laboratory for a fingerprick for

screening thick blood smear (using standard Giemsa staining). Thick blood

slides will be read and counted by the laboratory technicians. The parasite

density of positive screening thick blood smears will be estimated by counting

the number of asexual parasites per 200 leukocytes, assuming a leukocyte

count of 8,000/μl. Smears will be considered negative when examination of 100 high-power fields does not reveal parasites. Counts will be performed by two microscopists; discrepant readings will be resolved by a third reader. Gametocytes will be recorded as present or absent. Thin blood smears will be evaluated for parasite species.

(ii) Hemoglobin measure

Hemoglobin will measured from finger prick samples using a portable spectrophotometer (HemoCue, Ängelhom, Sweden).

(iii) In vitro test:

Patients with parasitemia of 4000/ul or more will be asked to

give 5ml of venous blood for in vitro test. The isotopic microtest or HRPII test

will be applied to determine the IC50 of piperaquine, lumefantrine, quinine

and DHA.

(iv) Molecular markers analysis:

Whenever blood will be collected, 4 drops will be placed onto filter paper,

labeled, air-dried, and stored in sealed plastic bags at ambient temperature.

Parasite DNA will subsequently be extracted using the Chelex extraction method.

We will use PCR techniques to determine new infection from recrudescence

and putative molecular markers of drug resistance. We will also analyze the

molecular markers associated with treatment failure and in vitro resistance, in particular polymorphisms in the P. falciparum pfcrt and pfmdr1 genes.

(v) Pharmacokinetic analysis

Plasma piperaquine concentrations will be determined by a validated sensitive and selective liquid chromatographic tandem mass-spectrometric (LC-MS/MS) method in the Pharmacology laboratory at Mahidol Oxford Tropical Medicine Research Unit in Bangkok.

# DATA COLLECTION AND MANAGEMENT

## *1. Data management*

All clinical data will be recorded onto standardized case record forms by study clinicians. Laboratory data will be recorded in a laboratory record book by the study laboratory technicians and then transferred to the case record forms by the study clinicians. Data will be transferred from the case record forms into a computerized database (EPI INFO 6.04) by data entry personnel and will be double entered to verify accuracy of entry. Two back-up files of the database will be stored on compact discs after each data entry session. For quality control, check programs will be written into the database to limit the entry of incorrect data and ensure entry of data into required fields.

## *2. Data quality assurance and monitoring*

All members of the study team will be educated in the study protocol prior to the onset of the trial. The study clinicians will complete case record forms at each patient visit. These forms will be reviewed by the study coordinator and site supervisors from the core facility for completeness and accuracy. For quality control of thick blood smear slide readings, expert microscopists will repeat the reading of all slides. All discrepant slide readings will be resolved based on the results of a 3^rd^ reading. Study group meetings will be conducted by the coordinator once a week to assess progress of the study, address any difficulties, and provide performance feedback to the members of the study group. In addition members from the core facility will make regular visits to active study sites as needed.

## *3. Records*

Case record forms will be provided for each subject. Participants will be identified by their initials and study identification number on the case record form. Patient names will not be entered into the computerized database. All patient record forms will be kept in a secure filing cabinet in the study clinics. All corrections will be made on case record forms by striking through the incorrect entry with a single line and entering the correct information adjacent to it. The correction will be initialed and dated by the investigator. Any requested information that is not obtained as specified in the protocol will have an explanation noted on the case record form as to why the required information was not obtained. Additional records will be kept in the clinical and laboratory record books at the core facility in Bobo-Dioulasso. The investigators will allow all requested monitoring visits, audits or reviews.

***4. Statistical analysis and sample size***

We will search for associations between diminished drug levels of piperaquine and clinical outcomes. Available resources will allow us to study 300 patients who complete study follow-up. Estimating a 10% loss to follow-up over 42 days, we will enroll 330 patients of whom 200 will have age range from 2 to 10 years. In our recent study at the same sites, the unadjusted 42 day risk of treatment failure with DP was 7.5%. Unadjusted failures are relevant in this analysis, as low levels of piperaquine may be associated with either recrudescence or new infections emerging during the elimination phase of piperaquine. It is estimated, based on our recent study that studying 300 patients will identify 22 unadjusted treatment failures. This sample size will provide the power to identify a 3-fold difference in unadjusted treatment efficacy between the lowest piperaquine level quartile and the remainder of subjects.

**PROTECTION OF HUMAN SUBJECTS**

## *1. Institutional Review Board (IRB) review and informed consent*

This protocol and the informed consent documents, including any additional educational or recruitment material, will be reviewed and approved by the Centre Muraz IRB, and an equivalent document will be reviewed by the UCSF Committee for Human Research, both before the trial begins. Any amendments or modifications to this material will be reviewed and approved by these bodies prior to implementation.

## *2. Risks and discomforts*

### Privacy

Care will be taken to protect the privacy of subjects, as described in this protocol. However, there is a risk that others may inadvertently see patients’ medical information, and thus their privacy may be compromised.

### Fingerprick blood draws

Risks include pain, transient bleeding and soft-tissue infection.

### Risk of dihydroartemisinin-piperaquine (DP)

### DP is an artemisinin-containing fixed-combination drug developed in China. Recent randomized clinical trials in Cambodia, Vietnam, Thailand, Uganda, and Burkina Faso indicate excellent tolerability and high cure rates against multi-drug resistant falciparum malaria. Artemisinin derivatives such as dihydroartemisinin have been used safely in large numbers of patients with uncomplicated or severe malaria. Piperaquine has been used less widely, but it has been a standard antimalarial drug in China since the 1970s. Data from in vitro and animal studies suggest that it is as potent as chloroquine but less toxic. [12, 14] In the first human studies of piperaquine, the main side effects were mild headache, listlessness, nausea, and dizziness. [15]In a study of the safety and efficacy of DP in 106 Cambodian children and adults with uncomplicated malaria, adverse events were uncommon (< 5%), mild, short lived, and difficult to distinguish from symptoms of malaria (anorexia, nausea, vomiting, abdominal pain, diarrhea, and dizziness). [16]In a safety evaluation of DP in 62 Cambodian children and adults with malaria, DP was found to be safe and well tolerated with no evidence of clinically significant postural hypotension, QTc prolongation, or propensity for hypoglycemia.[17] In a clinical trial of DP in 166 Vietnamese patients with uncomplicated malaria, 3% of patients reported minor adverse events, mostly transient nausea, which were self limited and resolved with the abatement of fever.[18] In a dose-optimization clinical trial of DP in 487 children and adults from Thailand with uncomplicated malaria, DP was well tolerated, with a low incidence of mild adverse events, which were mainly upper gastrointestinal and were similar to those reported in other studies.[19] In a clinical trial of DP in 331 children and adults from Thailand with uncomplicated malaria, DP was well tolerated, with a low incidence of mild side effects and no serious adverse events felt to be related to the study drug. [20]In 211 patients treated with DP in Uganda, the drug was well tolerated, and all serious adverse events were judged unrelated to the study drug [21]. In 187 patients treated with DP for malaria in Burkina Faso, the drug was well tolerated, with no serious adverse events (Zongo, et al., submitted). DP is now in routine use in Vietnam with no reports of serious adverse events (although limited resources are available for pharmacovigilance).

## *3. Compensation*

The patients/patients’ families will receive reimbursement for transportation costs to and from the clinic. In addition, all clinic visits, antimalarial medication, and the evaluation and treatment for some routine medical problems encountered during follow-up will be provided free of charge. Medical care that the patient receives which is unrelated to malaria will remain the primary responsibility of the patient, parent or guardian, although routine medical problems will generally be managed by the study at no cost to the patient.

## *4. Consent procedures*

All screening interviews will be conducted in the native language of the patients by the study personnel (with a translator if necessary). Consent forms will be provided to the parents or guardians for their review. Study clinicians will seek formal consent. The parents or guardians will be asked to sign consent to participate in the research study. The informed consent will describe the purpose of the study, the procedures to be followed, and the risks and benefits of participation. If a parent or guardian is unable to read or write, his/her fingerprint will be used in substitute for a signature, and a signature from a witness to the informed consent discussion will be obtained. Parents or guardians will be informed that participation of their child(ren) in the study is completely voluntary and that they may withdraw from the study at any time.

## *5. Alternatives*

Individuals whose parents or guardians choose not to participate in this study will not be enrolled. They will receive standard care for medical problems as they arise at the government health dispensaries or other medical facilities.

## *6. Confidentiality of records*

Patients, parents and guardians will be informed that participation in a research study may involve a loss of privacy. All records will be kept as confidential as possible. Patients will be identified primarily by their study number and patient names will not be entered into the computerized database. No individual identities will be used in any reports or publications resulting from the study.

# TIMETABLE

# Once all of the appropriate approvals are obtained enrollment of patients to be included in the analyses will begin and we will need 6-7 (June-December) months to complete the enrollment and the follow up.

**BUDGET**

**Estimated budget (for 200 children)**

- US$500.00 per child x 200 = ….euros US$100000.00
- In vitro and molecular (lab work) = ….euros US$9498.00
- Equipment & supplies = ….euros US$12000.00

***S/Total …. euros US$121498.00***

- Overhead (10%) ….. euros US$12149.80

**TOTAL …. euros US$133647.80**

**References**

1. Campbell CC. Challenges facing antimalarial therapy in Africa. J Infect Dis 1991;163:1207-11

2. Trape JF, Pison G, Preziosi MP, et al. Impact of chloroquine resistance on malaria mortality. C R Acad Sci III 1998;321:689-97

3. Guerin PJ, Olliaro P, Nosten F, et al. Malaria: current status of control, diagnosis, treatment, and a proposed agenda for research and development. Lancet Infect Dis 2002;2:564-73

4. White NJ. Antimalarial drug resistance. J Clin Invest 2004;113:1084-92

5. Piola P, Fogg C, Bajunirwe F, et al. Supervised versus unsupervised intake of six-dose artemether-lumefantrine for treatment of acute, uncomplicated Plasmodium falciparum malaria in Mbarara, Uganda: a randomised trial. Lancet 2005;365:1467-73

6. van den Broek I, Kitz C, Al Attas S, Libama F, Balasegaram M and Guthmann JP. Efficacy of three artemisinin combination therapies for the treatment of uncomplicated Plasmodium falciparum malaria in the Republic of Congo. Malar J 2006;5:113

7. Zongo I, Dorsey G, Rouamba N, et al. Artemether-lumefantrine versus amodiaquine plus sulfadoxine-pyrimethamine for uncomplicated falciparum malaria in Burkina Faso: a randomised non-inferiority trial. Lancet 2007;369:491-8

8. (2005). PNdLclP. Rapport de la réunion de consensus sur le changement de politique de traitement du paludisme au Burkina Faso. Ouagadougou. 21-23 Février 2005; 29 pp.

9. Zongo I, Dorsey G, Rouamba N, et al. Amodiaquine, sulfadoxine-pyrimethamine, and combination therapy for uncomplicated falciparum malaria: a randomized controlled trial from Burkina Faso. Am J Trop Med Hyg 2005;73:826-32

10. Dorsey G SS, Clark TD, Njama-Meya D, Nzarubara B, Maiteki-Sebuguzi C, Dokomajilar C, Kamya MR, Rosenthal PJ. Combination therapy for the treatment of uncomplicated falciparum malaria in a cohort of Ugandan children: a longitudinal randomized trial. JAMA, in press.

11. Karema C, Fanello CI, van Overmeir C, et al. Safety and efficacy of dihydroartemisinin/piperaquine (Artekin) for the treatment of uncomplicated Plasmodium falciparum malaria in Rwandan children. Trans R Soc Trop Med Hyg 2006;100:1105-11

12. Te-Yu Hung, Timothy M. E. Davis, Kenneth F. Ilett, et al. Population pharmacokinetics of piperaquine in adults and

children with uncomplicated falciparum or vivax malaria. British Journal of Clinical Pharmacology

2003;3:253-262

13. World Health Organization. Susceptibility of Plasmodium falciparum to antimalarial drugs. WHO/HTM/MAL/2005.1103

14. Zhang KY ZJ, Wu Z, Huang QL. [Susceptibility of Plasmodium falciparum to chloroquine, piperaquine, amodiaquine, mefloquine and quinine with in vitro microtechnique in Hainan Island]. Zhongguo Ji Sheng Chong Xue Yu Ji Sheng Chong Bing Za Zhi 1987;5:165-9

15. Chen L QF, Zhou YC. Field observations on the antimalarial piperaquine. Chin Med J (Engl) 1982;95:281-6

16. Denis MB, Davis TM, Hewitt S, et al. Efficacy and safety of dihydroartemisinin-piperaquine (Artekin) in Cambodian children and adults with uncomplicated falciparum malaria. Clin Infect Dis 2002;35:1469-76

17. Karunajeewa H, Lim C, Hung TY, et al. Safety evaluation of fixed combination piperaquine plus dihydroartemisinin (Artekin) in Cambodian children and adults with malaria. Br J Clin Pharmacol 2004;57:93-9

18. Tran TH, Dolecek C, Pham PM, et al. Dihydroartemisinin-piperaquine against multidrug-resistant Plasmodium falciparum malaria in Vietnam: randomised clinical trial. Lancet 2004;363:18-22

19. Ashley EA, Krudsood S, Phaiphun L, et al. Randomized, controlled dose-optimization studies of dihydroartemisinin-piperaquine for the treatment of uncomplicated multidrug-resistant falciparum malaria in Thailand. J Infect Dis 2004;190:1773-82

20. Ashley EA, McGready R, Hutagalung R, et al. A randomized, controlled study of a simple, once-daily regimen of dihydroartemisinin-piperaquine for the treatment of uncomplicated, multidrug-resistant falciparum malaria. Clin Infect Dis 2005;41:425-32

21. Kamya M, Yeka A, Bukirwa H, Lugemwa M, Rwakimari JB, Staedke SG, Talisuna AO, Greenhouse B, Nosten F, Rosenthal PJ, Wabwire-Mangen F, Dorsey G. Artemether-lumefantrine Versus Dihydroartemisinin-piperaquine for Treatment of Uncomplicated Falciparum Malaria: A Randomized Clinical Trial at a High Transmission Site in Uganda. PLoS Clin Trials, in press.
